# Validation of Computed Tomography-based Attenuation Correction of Deviation between Theoretical and Actual Values in Four Computed Tomography Scanners

**DOI:** 10.7508/aojnmb.2016.02.004

**Published:** 2016

**Authors:** Nobuhiro Yada, Hideo Onishi

**Affiliations:** 1 Biological Systems Sciences Program, Graduate School of Comprehensive Scientific Research, Prefectural University of Hiroshima, Hiroshima, Japan; 2 Department of Radiology, Shimane University Hospital, Izumo, Japan

**Keywords:** Attenuation coefficient, Bilinear scaling, CTAC, Effective atomic number

## Abstract

**Objective(s):**

In this study, we aimed to validate the accuracy of computed tomography-based attenuation correction (CTAC), using the bilinear scaling method.

**Methods:**

The measured attenuation coefficient (μ_m_) was compared to the theoretical attenuation coefficient (μ_t_), using four different CT scanners and an RMI 467 phantom. The effective energy of CT beam X-rays was calculated, using the aluminum half-value layer method and was used in conjunction with an attenuation coefficient map to convert the CT numbers to μ_m_ values for the photon energy of 140 keV. We measured the CT number of RMI 467 phantom for each of the four scanners and compared the μ_m_ and μ_t_ values for the effective energies of CT beam X-rays, effective atomic numbers, and physical densities.

**Results:**

The μ_m_ values for CT beam X-rays with low effective energies decreased in high construction elements, compared with CT beam X-rays of high effective energies. As the physical density increased, the μ_m_ values elevated linearly. Compared with other scanners, the μ_m_ values obtained from the scanner with CT beam X-rays of maximal effective energy increased once the effective atomic number exceeded 10.00. The μ_m_ value of soft tissue was equivalent to the μ_t_ value. However, the ratios of maximal difference between μ_m_ and μ_t_ values were 25.4% (lung tissue) and 21.5% (bone tissue), respectively. Additionally, the maximal difference in μ_m_ values was 6.0% in the bone tissue for each scanner.

**Conclusion:**

The bilinear scaling method could accurately convert CT numbers to μ values in soft tissues.

## Introduction

Most single-photon emission computed tomography (SPECT) and positron emission tomography (PET) studies have utilized computed tomography (CT)-based attenuation correction (CTAC). CTAC can reflect the attenuation of gamma rays for the body and acquisition bed and facilitate the construction of attenuation coefficient maps (μ maps) for individual patients.

Ishii et al. recommended the use of CTAC to obtain accurate regional cerebral blood flow SPECT images (1). Moreover, in a previous study, myocardial perfusion images with CTAC exhibited a uniform radioactivity distribution in both 180° and 360° arc acquisitions (2). CTAC applies to attenuation correction by conversion from a CT number to an attenuation coefficient (μ value). However, the photon energy of CT beam X-rays (CT X-rays) is lower than specific photon energies of gamma rays, used in clinical nuclear medicine, and the attenuation effects differ with variations in energy.

It is necessary to convert the attenuation data acquired via CT scan to match the energy of the radionuclide used in SPECT acquisitions. This is typically accomplished by using the bilinear scaling method, relating the μ value at the desired energy to the CT number measured at the effective energy of CT X-rays (3). The bilinear scaling method determines μ values via bilinear calibration lines, which are delimited at a CT number of zero Hounsfield Units (HU) and are most commonly used in PET/CT scanners.

Ay et al. compared six energy-mapping approaches and reported that the bilinear calibration curve technique yielded acceptable attenuation maps in a PET study (4). The bilinear scaling method is widely used in SPECT studies, considering its association with highly accurate conversions in high construction elements.

Reportedly, the effective energies of CT X-rays from different CT systems vary, even when they are set at the same tube voltage (5). The interaction process is influenced by the effective atomic number, physical density, and effective energy of CT X-rays (6). Therefore, μ value is differentially affected by various CT systems and variations in the construction elements of different tissues.

In this study, we validated the accuracy of the conversion of CT numbers to μ values, particularly with regard to differences in the effective energies of CT X-rays and CT scanners in SPECT/CT studies. Also, the aim of this study was to validate the accuracy of CTAC, based on the bilinear scaling method.

The measured attenuation coefficients (μ_m_ values) were compared with the CT numbers, effective atomic numbers, and physical densities, using an RMI 467 tissue characterization phantom (RMI 467 phantom). The μ_m_ values were then compared to theoretical attenuation coefficients (μ_t_ values) via four different multi-detector CT (MDCT) scanners.

## Methods

This study qualitatively compared the theoretical and measured μ values, obtained from four different MDCT scanners, using the bilinear scaling method and identified materials. We used μ maps to evaluate the μ_m_ values, derived from each scanner. The μ maps were generated by converting the CT number distribution to the attenuation coefficient distribution. These maps were derived from CT images and effective energies of CT X-rays, using the bilinear scaling method and an image processing workstation.

The four MDCT scanners used in this study were as follows: a 16-slice Discovery NM/CT 670 Pro-scanner Brightspeed 16 (G-CT) (GE Healthcare, WI, USA), a 320-slice Aquilion ONE CT scanner (T-CT) (Toshiba Medical Systems, Tokyo, Japan), a 64-slice Brilliance CT 64 scanner (P-CT) (Philips Healthcare, Cleveland, OH, USA), and a 24-slice SOMATOM Sensation Open ICT scanner (S-CT) (Siemens, Erlangen, Germany).

We imported each CT image into an image processing workstation (GE Xeleris 3.0), and the μ values were converted, using the same workstation. Radiation exposure was measured with Accu-Dose Model 2186 and 10×6-3 CT ionization chamber dosimeter (Radcal Corporation, Monrovia, CA, USA).

### Tissue characterization phantom

The RMI 467 tissue characterization phantom (RMI 467 phantom), which was 330 mm in diameter, was positioned vertically to the bed and the rods were inserted inside the phantom. We evaluated the CT number and μ value in ten rods. Table 1 shows the elemental compositions of tissue substitutes with the rod of RMI 467 phantom. The RMI 467 phantom consisted of water-equivalent materials, with a physical density of 1.02 g/cm^3^ and an electron density of 0.99.

**Table 1:** The elemental composition of the materials in the RMI 467 phantom

Weight Percentage									
Rod No	Material	H	C	N	O	Mg	Si	Cl	Ca
1	Lung - LN300	8.5	59.3	2.0	18.1	11.2	0.8	0.1	0.0

2	Lung - LN450	8.5	59.5	2.0	18.1	11.2	0.6	0.1	0.0

3	AP6 Adipose	9.1	72.2	2.3	16.3	0.0	0.0	0.1	0.0

4	BR12 Breast	8.7	70.0	2.4	17.9	0.0	0.0	0.1	1.0

5	CT Solid Water	8.1	67.2	2.4	19.8	0.0	0.0	0.1	2.3

6	LV1 Liver	11.0	67.0	2.5	20.0	0.0	0.0	0.1	2.3

7	SR2 Brain	10.8	72.5	1.7	14.9	0.0	0.0	0.1	0.0

8	CB2 - 30% CaCO_3_	6.7	55.6	2.1	25.6	0.0	0.0	0.1	12.0

9	CB2 - 50% CaCO_3_	4.8	41.6	1.5	32.0	0.0	0.0	0.1	20.0

10	SB3 Bone Cortical	3.4	31.4	1.8	36.5	0.0	0.0	0.0	26.8

**Figure 1:**
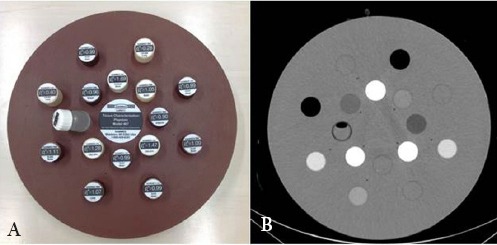
Front view of (a) the RMI 467 tissue characterization phantom and (b) the CT image

The rods were cylindrical, encompassing X-ray attenuation ranges of human tissues, specifically 0.28-0.45, 0.94-1.05, and 1.34-1.82 g/cm^3^ for the lung, soft tissue, and bone, respectively. The rods were placed in an arrangement designed to reduce the effects of artifacts from high construction elements (Figure 1). The conditions applied in the RMI 467 phantom (i.e., elemental composition, physical density, and electron density) were based on the data, generated by the Texas Medical Center, Texas, USA (7).

### Data acquisition and processing

CT datasets were acquired, using G-CT, T-CT, P-CT, and S-CT scanners with conditions including tube voltages of 140, 120, 100, and 80 kV, a tube current-time product of 400 mAs, and a field of view of 500 mm. The CT datasets were reconstructed, using a filtered back projection algorithm with a soft kernel, soft (G-CT), FC11 (T-CT), Standard A (P-CT), and B20s (S-CT) in 5-mm-thick axial images of 512×512 pixels.

The μ maps were generated, using the measured effective energies of CT X-rays, corresponding to each CT scanner. Then, the CT images were resized from 512×512 to 128×128 matrices, and each CT number was converted to a μ value, based on the National Institute of Standards and Technology (NIST) data in the Xeleris workstation 3.0 (8). The CT numbers and μ values were measured in regions of interest (ROIs) on the rods.

### Evaluation

We compared μ values with CT numbers, effective atomic numbers, and physical densities. We also measured the CT numbers, yielded by the CT X-rays of different effective energies and evaluated the CT numbers and μ values, obtained from four different CT scanners.

The CT numbers corresponding to CT X-rays of different energies were verified, using the G-CT scanner. Then, the μ_m_ and μ_t_ values derived from the four scanners were compared. The effective energies of CT X-rays were previously measured via the aluminum half-value layer method (9) and were used to convert the μ_m_ values.

### CT numbers of CT X-rays with different effective energies

The effective energies of CT X-rays from the scanners are presented in Table 2. The CT number of each rod was verified with the effective atomic number and physical density at effective energies of 64 keV (140 kV), 58 keV (120 kV), 53 keV (100 kV), and 47 keV (80 kV), using the G-CT scanner. The average CT number was derived from circular ROIs (diameter: 80% of the rod; 600 pixels) on each rod. We assessed the average CT number of CT X-ray effective energies in each rod.

**Table 2:** Parameters for reconstruction and attenuation correction maps in four computed tomography scanners

	T-CT (120 kV)	S-CT (120 kV)	G-CT (140 kV/ 120 kV/ 100 kV/ 80 kV)	P-CT (120 kV)
Effective energy (keV)	52.0	57.6	64.1 / 58.1 / 53.3 / 47.0	65.6

μ _water, x_ (cm^-1^)	0.222	0.210	0.201 / 0.210 / 0.220 / 0.238	0.198

μ _bone, x_ (cm^-1^)	0.764	0.639	0.559 / 0.639 / 0.740 / 0.923	0.539

Equation 1 was used to determine the effective atomic number (*Z_eff_*):


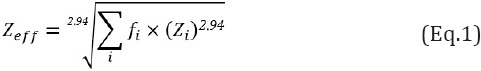


Where *f_i_* is the ratio of the electron number, and *Z_i_* denotes the atomic number [^10^]. The effective atomic number was used to evaluate the interaction processes.

### CT numbers and μ values derived from four different CT scanners

Table 3 shows the theoretical attenuation coefficients, which were calculated using the mass attenuation coefficient, based on the NIST data. The calculated attenuation coefficient was regarded as the μ_t_ value in this study. The relationship between the mass attenuation coefficient (μ/ρ) and the photon cross-section was defined by Equation 2:

**Table 3:** Linearity rod data

Rod No	Material	Effective Atomic Number	Physical Density	Electron Density	Liner Attenuation Coefficient

			(g/cm^3^)	Relative to Water	(cm^-1^)
1	Lung - LN300	7.62	0.28	0.28	0.040

2	Lung - LN450	7.60	0.45	0.40	0.060

3	AP6 Adipose	6.32	0.94	0.90	0.138

4	BR12 Breast	6.90	0.98	0.96	0.145

5	CT Solid Water	7.60	1.02	0.99	0.148

6	LV1 Liver	7.60	1.09	1.07	0.151

7	SR2 Brain	6.23	1.05	1.05	0.160

8	CB2 - 30% CaCO_3_	10.6	1.34	1.28	0.175

9	CB2 - 50% CaCO_3_	12.22	1.56	1.47	0.183

10	SB3 Bone Cortical	13.30	1.82	1.69	0.193


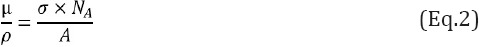


Where ρ is the physical density, σ is the photon cross-section, *N_A_* denotes the Avogadro constant, and *A* is the atomic weight. We calculated the μ_t_ value for a 140 keV photopeak in rod No.7, which consisted of the following elements: H (10.8%), C (72.5%), N (1.7%), O (14.9%), and Cl (0.1%) (Table 1).

For each photon cross-section, the NIST yielded the following values at 140 keV photopeak: 0.270, 0.138, 0.138, 0.139, and 0.155 cm^2^/g for H, C, N, O, and Cl, respectively; the μ_t_ value was 0.160 cm^-1^ in rod No. 7. Also, the μ_t_ value for the 140 keV photopeak was calculated for each rod.

The μ_m_ value determined by the bilinear scaling method was defined by the Xeleris workstation 3.0, using equations 3 and 4:


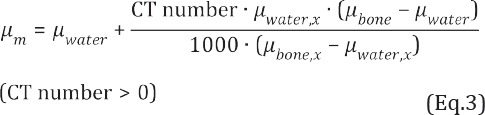



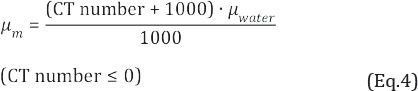


Where μ_water_ and μ_bone_ are the linear attenuation coefficients of water and bone for the gamma ray energy, respectively, and μ_water, x_ and μ_bone, x_ denote the linear attenuation coefficients of water and bone for the effective energies of CT X-rays emitted from the scanners, respectively.

The μ_m_ and μ_t_ values were compared with the CT numbers, effective atomic numbers, and physical densities at a tube voltage of 120 kV for each of the four CT scanners. The μ_m_ values were calculated from circular ROIs (diameter: 80% of the rod; 60 pixels) on each rod on the μ map. The μ_m_ values were compared, using the Steel-Dwass test (P<0.05).

## Results

### CT numbers and effective energies of CT X-rays

**Figure 2:**
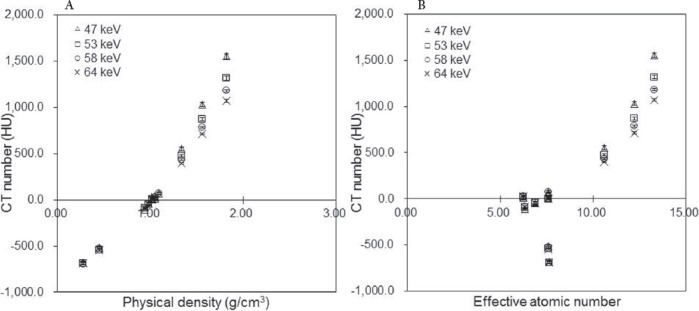
Mean CT number (HU) as a function of (a) physical density and (b) effective atomic number in the RMI 467 phantom at different effective CT X-ray energies (Δ47 keV, □53 keV, ο 58 keV, ×64 keV). Regions of interest (ROIs) were set on the rod regions in CT images, and the mean CT numbers were calculated

Figure 2 shows the mean values of CT numbers in the ROIs in each rod of the RMI 467 phantom for different effective CT X-ray energies, effective atomic numbers, and physical densities. At a physical density of < 1.34 g/cm^3^, the mean CT number increased as the effective energy decreased (Figure 2a). The mean CT number increased at 47 keV, compared with 64 keV. The differences between the mean CT numbers at physical densities of 0.28, 1.56, and 1.82 g/cm^3^ were 1.1%, 44.2%, and 45.1%, respectively. In terms of the lung tissue, the effective atomic number was not linearly related to dose (Figure 2b).

### CT numbers and μ values from the four different scanners

**Figure 3:**
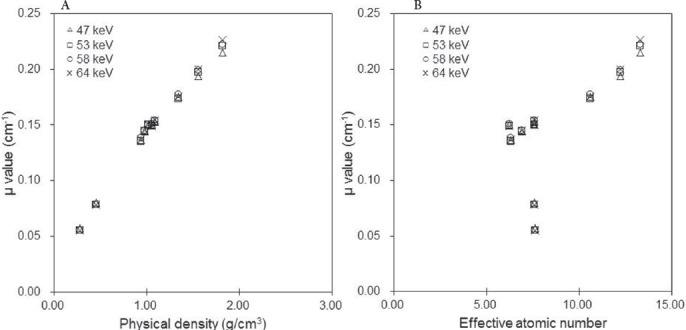
Attenuation coefficients (μ_m_ values; cm^-1^) as a function of (a) physical density and (b) effective atomic number (Δ47 keV, □53 keV, ο 58 keV, ×64 keV). Regions of interest (ROIs) were set on the rod regions in CT images (at a CT X-ray effective energy of 58 keV and a tube current time of 400 mAs) and copied to a μ_m_ map

Figure 3 and Table 4 show the μ_m_ values in each rod of RMI 467 phantom at each CT X-ray effective energy, effective atomic number, and physical density. The μ_m_ values increased in response to the rise in physical density (Figure 3a). In comparison with 47 keV, the μ_m_ values at 64 keV significantly differed by 7.4%, 5.9%, and 9.5% at physical densities of 0.28, 1.56, and 1.82 g/cm^3^, respectively (P<0.05).

**Table 4:** Comparisons of attenuation coefficient differences in G-CT

Rod No	Material	64 keV vs 58 keV	64 keV vs 53 keV	64 keV vs 47 keV	58 keV vs 53 keV	58 keV vs 47 keV	53 keV vs 47 keV
1	Lung - LN300	n.s.	n.s.	*	n.s.	*	*

2	Lung - LN450	n.s.	n.s.	*	n.s.	*	*

3	AP6 Adipose	n.s.	*	n.s.	*	*	n.s.

4	BR12 Breast	n.s.	n.s.	n.s.	n.s.	n.s.	n.s.

5	CT Solid Water	n.s.	n.s.	n.s.	n.s.	n.s.	n.s.

6	LV1 Liver	n.s.	n.s.	n.s.	n.s.	n.s.	n.s.

7	SR2 Brain	*	n.s.	n.s.	*	n.s.	n.s.

8	CB2 - 30% CaCO_3_	*	*	*	*	*	*

9	CB2 - 50% CaCO_3_	*	*	*	*	*	*

10	SB3 Bone Cortical	*	*	*	*	*	*

Steel-Dwass test *: p < .05, n.s.: not significant. 64 keV (140 kV), 58 keV (120 kV), 53 keV (100 kV), 47 keV (80 kV)

For the soft tissue range, the μ_m_ values at 47 and 64 keV were similar; the differences between the μ_m_ values at 47 keV and 64 keV were < 1.5%. Also, the difference ratio of μ_m_ values at 58 keV was 11% for the soft tissue and 21% for the bone tissue.

**Figure 4:**
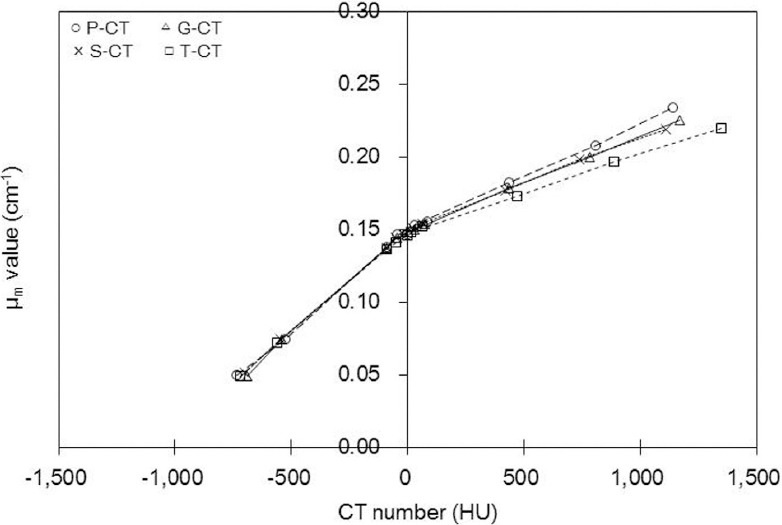
Attenuation coefficients (μ_m_ values; cm^-1^) as a function of the mean CT number derived from each of the four scanners (ο P-CT, ΔG-CT, ×S-CT, □T-CT). The differences between the bilinear calibration lines, especially between the P-CT and T-CT scanners, tended to increase as indicated by the slope of the regression line at HU > 0. They were y=7.4×10^-5^+0.149 [ο P-CT], y=6.6×10^-5^+0.149 [ΔG-CT], y=6.6×10^-5^+0.149 [×S-CT], and y=5.5×10^-5^+0.147 [□T-CT]

Figure 4 shows the μ_m_ values, derived from the mean CT numbers of the four CT scanners. The slope of the regression function in the CT number range below zero HU was equal in each scanner, whereas at CT numbers over zero HU, the corresponding values were 7.4×10^-5^, 6.6×10^-5^, 6.6×10^-5^, and 5.5×10^-5^ for the P-CT, G-CT, S-CT, and T-CT scanners, respectively.

**Figure 5:**
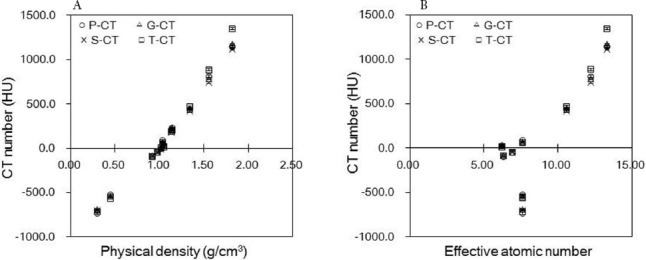
Mean CT numbers (HU) as a function of (a) physical density and (b) effective atomic number, derived by the RMI 467 phantom for the four investigated scanners (ο P-CT, ΔG-CT, ×S-CT, □T-CT)

Figure 5a shows the mean CT numbers for the rods as determined by each of the four scanners. For high construction elements, the mean CT number of the T-CT scanner was greater than that of other scanners. The difference ratios of the mean CT numbers, yielded by T-CT and S-CT scanners, were 2.8%, 1.9%, and 17.5% at physical densities of 0.28, 1.09, and 1.82 g/cm^3^, respectively.

**Figure 6:**
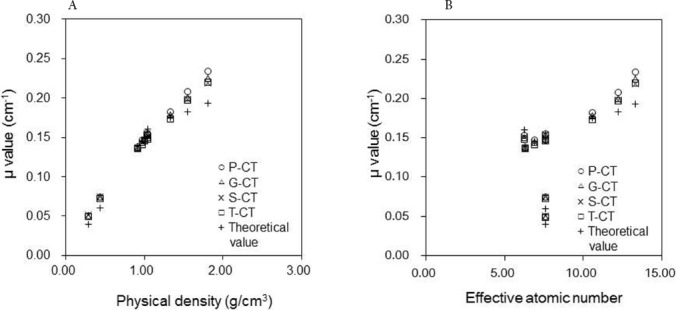
Attenuation coefficients (μ_m_ values; cm^-1^) as a function of (a) physical density and (b) effective atomic number in the RMI 467 phantom derived from the four investigated scanners (ο P-CT, ΔG-CT, ×S-CT, □T-CT, + theoretical value). Theoretical values are shown as μ values for the 140 keV photopeak, and the μ_m_ values for each scanner were converted via the measured effective energy of CT X-rays

As shown in Figure 6 and Table 5, in numerous comparisons, the μ_m_ value differed from the μ_t_ value, which was true for all the tested CT scanners. While the μ_m_ and μ_t_ values were similar in the soft tissue range, they tended to differ in the bone tissue range. The μ_m_ values derived from the P-CT scanner were 0.21 and 0.23 cm^-1^ at physical densities of 1.56 and 1.82 g/cm^3^, respectively, and the corresponding ratios of difference between μ_m_ and μ_t_ values were 14.0% and 21.5%, respectively.

**Table 5:** Comparisons of attenuation coefficient differences in four computed tomography scanners

Rod No	Material	G-CT vs T-CT	G-CT vs P-CT	G-CT vs S-CT	T-CT vs P-CT	T-CT vs S-CT	P-CT vs S-CT
1	Lung - LN300	n.s.	n.s.	n.s.	*	*	n.s.

2	Lung - LN450	*	n.s.	n.s.	*	*	n.s.

3	AP6 Adipose	n.s.	n.s.	n.s.	*	n.s.	n.s.

4	BR12 Breast	*	*	n.s.	*	*	*

5	CT Solid Water	*	n.s.	n.s.	n.s.	n.s.	n.s.

6	LV1 Liver	*	n.s.	n.s.	*	*	n.s.

7	SR2 Brain	*	*	*	*	*	*

8	CB2 - 30% CaCO_3_	*	*	*	*	*	*

9	CB2 - 50% CaCO_3_	*	*	*	*	*	*

10	SB3 Bone Cortical	*	*	*	*	*	*

Steel-Dwass test *: p < .05, n.s.: not significant

In the lung tissue range, at an effective atomic number of 7.60, the μ_m_ values derived from the P-CT were 0.05 and 0.07 cm^-1^ at physical densities of 0.28 and 0.45 g/cm^3^, respectively, and the corresponding ratios of difference between μ_m_ and μ_t_ values were 25.4% and 23.5%, respectively. As the effective energy of CT X-rays increased, the μ_m_ values were significantly elevated, and the differences between μ_m_ values derived from the T-CT and P-CT scanners were 2.0%, 4.1%, and 6.0% at physical densities of 0.28, 1.09, and 1.82 g/cm^3^, respectively (P<0.05).

## Discussion

By using the bilinear scaling method, CTAC could generate μ maps, regardless of the type of CT scanner used for SPECT/CT. The clinical advantages of CTAC have been previously reported (1, 2). In the present study, we validated the accuracy of μ values, using an RMI 467 phantom in four CT scanners.

In this CT study, attenuations were affected by the photoelectric effect and Compton scattering. Therefore, high CT numbers were associated with CT X-rays of low effective energy and high atomic number. Compared to other effective energies of CT X-rays, the mean CT numbers associated with the effective energies of CT X-rays significantly increased at 47 keV as the physical density and effective atomic number elevated, especially at effective atomic numbers greater than 10.00 (> 1.34 g/cm^3^) (Figure 2).

The obtained findings indicated that attenuation was dominated by a photoelectric effect, with increasing effective atomic number and decreasing effective energy of CT X-rays.

For the bone tissue, the mean CT number was more influenced by the effective energy of CT X-rays, compared to the lung or soft tissue. The μ_m_ values were associated with the CT numbers. Therefore, we concluded that a study involving μ values needed to incorporate these three tissue groups, i.e., lung, bone, and soft tissues, in order to represent the spectrum of human body tissues.

The difference ratio of μ_m_ values derived from the soft tissue was not greater than that of bone tissue, whereas the difference ratios of effective atomic numbers were 18% and 20% in soft and bone tissues, respectively (Figure 3b, Table 3). These results suggested that differences in the effective energy of CT X-rays had slight effects on μ_m_ values at low effective atomic numbers (soft tissue).

In the present study, the μ_m_ values at 47 keV decreased in the bone tissue due to the high μ_bone, x_ value (Figure 3a, Table 2), whereas the CT number increased (Figure 2). The μ_m_ values varied in different scanners, and the slope of regression function increased as the effective energy of CT X-rays increased (Figure 4).

In the present study, the μ_m_ values derived from the G-CT and S-CT scanners were almost equal, since the effective energies of CT X-rays were equivalent at 58 keV. The mean CT numbers yielded by the P-CT scanner did not significantly differ from those obtained by the G-CT or S-CT scanner (Figure 5). However, the mean values derived from the P-CT scanner were approximately 6% greater than those obtained by other scanners with regard to physical density (1.82 g/cm^3^) (Figure 6).

The present findings suggest that μ_m_ values were more affected by the μ_water, x_ and μ_bone,x_ values than the CT number. The μ_m_ values increased as the effective energies of CT X-rays elevated (Table 2, Equation 3); for all the scanners, the μ_m_ values were greater than the μ_t_ values. The interaction processes associated with X-ray energies dominated Compton scattering in the soft tissue and photoelectric effects in the bone tissue.

At the specific energy of 140 keV, the interaction process dominated Compton scattering in the bone tissue. Therefore, the accuracy of conversion for the bone tissue range yielded a lower accuracy. In this study, the calculated difference between μ_m_ and μ_t_ values for the lung tissue (0.28 g/cm^3^) was 22.9%. Since the lung tissue encompassed air, the μ_m_ values were of a lower physical density, compared to other investigated tissues. On the other hand, the effective atomic numbers of lung tissue were close to the soft tissue.

With regard to the effective atomic numbers of the soft tissue, the photoelectric effect on the CT X-ray effective energy was greater than the specific effective energy of 140 keV. Therefore, the μ_m_ values of lung tissue increased, compared to the μ_t_ values. The relationships between the mean CT numbers and μ values varied, since the relationship between the effective energy of CT X-rays and linear attenuation coefficients differed for each tissue.

Different scaling factors may be required for bone and soft tissues when converting CT images. LaCroix et al. suggested that the attenuation coefficient of bone tissue introduced a large error (11). Blankespoor et al. suggested that bilinear scaling could be used to convert CT images for the attenuation correction of SPECT data, as subsequently incorporated in a study by Kinahan et al. (12, 13).

Based on these findings, we can conclude that μ_m_ values for CT numbers above zero need to be converted via different scaling factors, unlike CT numbers below or equal to zero. However, in this study, μ_m_ values increased in the lung and bone tissues, indicating the low accuracy. Also, the errors in high construction elements were larger than those reported by Ay et al. (4) in a PET study.

In the present study, it was revealed that μ_m_ values derived from the T-CT scanner were close to μ_t_ values, whereas μ_m_ values obtained from the P-CT scanner were of a comparatively low accuracy (Figure 6). The CT numbers derived from the bone and soft tissues in the range of 50-60 keV were difficult to convert, since the ratio of the photoelectric effect and Compton scattering differed with variations in the effective energies of CT X-rays and effective atomic numbers.

We surmise that for the soft tissue, CTAC was accurate in each of the tested scanners. However, the accuracy of μ_m_ values for effective atomic numbers over 10.00 needs to be improved. This may be achieved via approaches designed to address the differences in the effective energies of CT X-rays.

In this study, the CT number depended on the X-ray beam quality and was consequently influenced by both the effective energy of CT X-rays and beam-hardening effects. The correction of beam-hardening effects when reconstructing the CT datasets improved the image quality and only resulted in slight changes in the CT numbers. The μ values derived from G-CT and S-CT scanners were not changed, since the effective energies of CT X-rays were equivalent, whereas their mean CT numbers varied. Therefore, we can deduce that beam-hardening correction is not significantly affected by the μ value.

## Conclusion

All four CT scanners tested in this study yielded a sufficiently high precision with regard to the accuracy of attenuation coefficient in the soft tissue ranges. However, the conversion of CT number to attenuation coefficient was poor in the ranges of lung and bone tissues. In all the scanners, the errors pertaining to high construction elements were caused by different interaction processes.

This study was supported by the Digital Image Scientific Research Meeting (Mihara, Hiroshima, Japan).
